# Complementary dynamic BH3 profiles predict co-operativity between the multi-kinase inhibitor TG02 and the BH3 mimetic ABT-199 in acute myeloid leukaemia cells

**DOI:** 10.18632/oncotarget.8742

**Published:** 2016-04-15

**Authors:** Monica Pallis, Francis Burrows, Jeremy Ryan, Martin Grundy, Claire Seedhouse, Amina Abdul-Aziz, Joan Montero, Anthony Letai, Nigel Russell

**Affiliations:** ^1^ Clinical Haematology, Nottingham University Hospitals, Nottingham, UK; ^2^ Tragara Pharmaceuticals, Carlsbad, CA, USA; ^3^ Department of Medical Oncology, Harvard Medical School, Boston, MA, USA; ^4^ Department of Haematology, University of Nottingham, Nottingham, UK

**Keywords:** BAD, NOXA, TG02, ABT-199, AML

## Abstract

Direct co-operation between sensitiser molecules BAD and NOXA in mediating apoptosis suggests that therapeutic agents which sensitise to BAD may complement agents which sensitise to NOXA. Dynamic BH3 profiling is a novel methodology that we have applied to the measurement of complementarity between sensitiser BH3 peptide mimetics and therapeutic agents. Using dynamic BH3 profiling, we show that the agent TG02, which downregulates MCL-1, sensitises to the BCL-2-inhibitory BAD-BH3 peptide, whereas the BCL-2 antagonist ABT-199 sensitises to MCL-1 inhibitory NOXA-BH3 peptide in acute myeloid leukaemia (AML) cells. At the concentrations used, the peptides did not trigger mitochondrial outer membrane permeabilisation in their own right, but primed cells to release Cytochrome C in the presence of an appropriate trigger of a complementary pathway. In KG-1a cells TG02 and ABT-199 synergised to induce apoptosis. In heterogeneous AML patient samples we noted a range of sensitivities to the two agents. Although some individual samples markedly favoured one agent or the other, in the group as a whole the combination of TG02 + ABT-199 was significantly more cytotoxic than either agent individually. We conclude that dynamic NOXA and BAD BH3 profiling is a sensitive methodology for investigating molecular pathways of drug action and complementary mechanisms of chemoresponsiveness.

## INTRODUCTION

The ability of therapeutics to kill leukaemia cells is in part dependent on the net effect of the interplay between pro-apoptotic and anti-apoptotic molecules. In particular, when sensitiser BCL-2 family proteins (such as BAD and NOXA) succeed in displacing activator proteins (such as BIM) from survival proteins (such as BCL-2 and MCL-1), the apoptotic effectors/executioners BAX and BAK activate mitochondrial outer membrane permeabilisation (MOMP) and the apoptotic cascade is set in motion [1, 2]. Thus the influence of BCL-2 family pro-survival molecules depends on the extent to which they can be neutralized by pro-apoptotic binding partners [3, 4].

When overexpressed in acute myeloid leukaemia cells, the survival proteins BCL-2 and MCL-1 confer chemoresistance [3, 5–7]. Pro-apoptotic BAX and BAK apoptosis effector function is thought to require the simultaneous neutralisation of all expressed pro-survival BCL family members, such that if MCL-1 is inhibited, BCL-2 may prevent apoptosis and vice versa [3, 8, 9]. This relationship was shown at the mechanistic levels by the complementary apoptotic function of the two sensitiser proteins NOXA (which targets the labile BCL-2 family survival proteins MCL-1, BCL2A1 and BCL2A10) and BAD (which targets BCL-2 and BCL-X_L_). NOXA and BAD together were toxic in a cellular assay where neither was effective individually [10].

**Figure 1:**
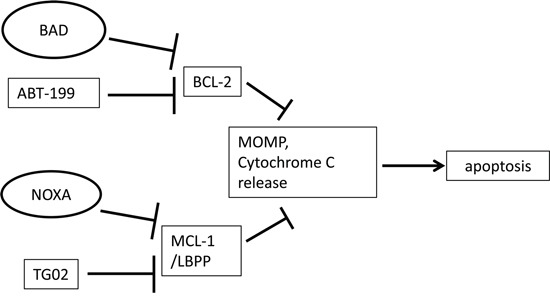
Schematic diagram In this simplified scenario, TG02 as well as endogenous NOXA suppress the pro-survival function of MCL-1 and maybe of additional labile BCL-2 family pro-survival proteins (LBPP) [17, 20, 40]. ABT-199 and endogenous BAD suppress the pro-survival function of BCL-2 [14, 20, 40]. Mitochondrial outer membrane permeabilisation (MOMP), cytochrome C release and subsequent apoptosis result from antagonism of pro-survival proteins. In some cell types, MOMP may arise from targeting either BCL-2 or MCL-1 alone, whereas some cells might require more than one survival molecule to be suppressed.

Several researchers have indicated that an agent which targets BCL-2 can synergise with an agent that targets MCL-1 [11–13]. In the current report, we demonstrate roles for NOXA and BAD in this type of dual dependency. We build upon the complementary function described for NOXA and BAD and investigate whether a chemotherapeutic agent that mimics the MCL-1 sensitising role of NOXA will co-operate with a BAD-BH3 peptide to trigger MOMP. Likewise we investigate whether an agent that mimics the BCL-2 sensitising role of BAD will co-operate with a NOXA-BH3 peptide to trigger MOMP (Figure 1 – schematic diagram). Finally we investigate the effects of using the two agents together to target AML cells.

ABT-199 is a small molecule BH3 mimetic, selective for BCL-2 and orally bioavailable [14, 15]. TG02 is a novel multi-kinase inhibitor which exerts greatest activity against the cyclin dependent kinase CDK9 (IC_50_ 3nM) [16]. CDK9 is permissive for transcription by phosphorylation of RNA polymerase II on serine 2 (RPIIS2), and in AML cells TG02 treatment causes rapid RPIIS2 dephosphorylation, such that RNA synthesis is strongly inhibited [16–18] and proteins with a short half-life, such as MCL-1, are rapidly downregulated [16, 17]. Our findings indicate the ability of TG02 to co-operate with BAD, the ability of ABT-199 to co-operate with NOXA and the ability of the two chemotherapeutic agents to co-operate with each other to induce cytochrome C release and apoptosis in AML cells.

## RESULTS

### TG02 and ABT-199 have complementary BH3 profiles

**Figure 2:**
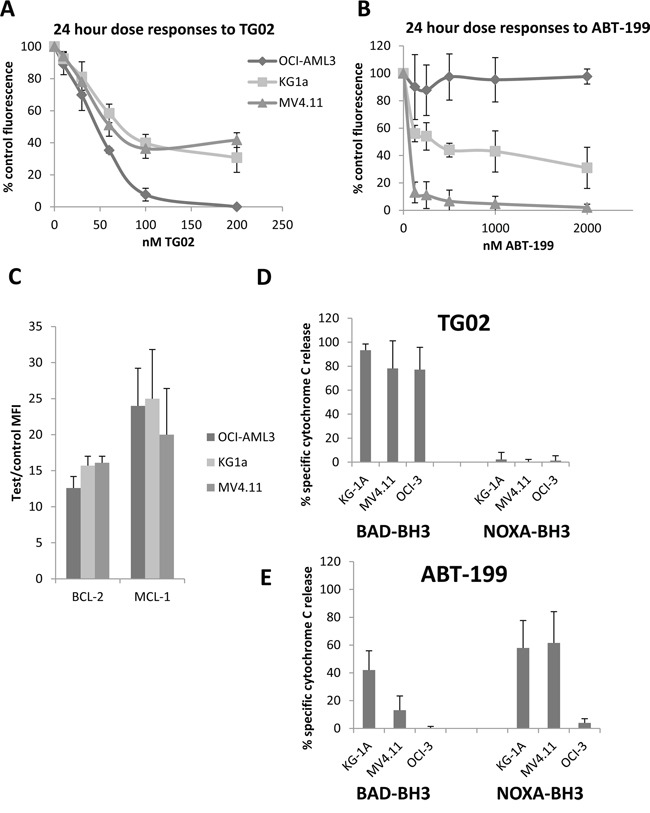
Responses to TG02 and ABT-199 and complementary BH3 profiles in KG-1a, OCI-AML3 and MV4.11 cell lines **A-B**. Cells were cultured at 5 X 10^5^/ml for 24 hours with the indicated concentrations of compounds and responses were measured by alamar blue assay. **C**. BCL-2, and MCL-1 were measured by flow cytometry. MFI = mean fluorescence intensity. **D-E**. Cells were treated for 4 hours with 100 nM TG02 or 500 nM ABT-199, permeabilised and treated for 1 hour with 23 μM NOXA-BH3 or with BAD-BH3 (0.3 μM in KG-1a and MV4.11, 3 μM in OCI-AML3) before fixation and labelling with Cytochrome C antibody for flow cytometry. The percentage of cells which had released cytochrome C, a measure of mitochondrial outer membrane permeabilisation, was measured on fluorochrome/side scatter dot plots. Adjustments for peptide-induced cytochrome C release in untreated cells were made by calculating agent-specific release according to the formula in the Methods section. All experimental results are mean ± SD of 3-4 independent assays.

To select suitable cells for our experiments we first established dose responses to the agents individually in three cell lines – MV4.11, KG-1a and OCI-AML-3. Responses were evaluated at 24 hours by alamar blue assay. All 3 cell lines were sensitive to nanomolar concentrations of TG02 (Figure 2A), but OCI-AML-3 are all eradicated, whereas MV4.11 and KG1a curves flatten with approximately 40% of cells remaining viable. OCI-AML3 cells were highly resistant to ABT-199 (Figure 2B); MV4.11 were sensitive and KG1a had intermediate sensitivity. BCL-2 and MCL-1 protein expression was similar in all three cell lines (Figure 2C).

Exposure of cells to drugs has been shown to reduce the concentration of BIM-BH3 peptide needed to induce mitochondrial outer membrane permeabilisation (MOMP) in sensitive samples [19]. BAD and NOXA are sensitiser BH3 proteins which can trigger MOMP and apoptosis by releasing BIM from anti-apoptotic BCL-2 family proteins. BAD specifically releases BIM from BCL-2, whereas NOXA targets MCL-1 [20]. Direct co-operation between BAD and NOXA in inducing apoptosis has been reported [10], suggesting that therapeutic agents which sensitise to BAD may complement agents which sensitise to NOXA, (Figure 1), likely through increasing the BIM released. To investigate this further, we used BH3 profiling, a technique which measures cytochrome C release in response to peptides derived from BH3-only pro-apoptotic family members [2, 21]. The technique is illustrated in Supplementary Figure 1. Loss of MCL-1 might be expected to release pro-apoptotic proteins which transfer to the remaining BCL-2 or BCLX_L_, and can be detected by increased sensitivity to their antagonist peptide BAD-BH3 [22]. We found that TG02 triggered cells for a nearly complete response after priming with BAD-BH3, but not NOXA-BH3 (Figure 2D). In KG1a cells, the specific RNA Polymerase II (RPII) inhibitor 5,6-dicholoro-1-β-D-ribofuranoslybenzimidazole (DRB) was used at the previously determined IC_50_ [18] to determine whether the BAD-priming effect was generic to RPII inhibition and this was found to be the case (P<0.001, Supplementary Figure 2). ABT-199, in contrast to TG02, triggered MV4.11 and KG-1a cells to respond to the MCL-1 sensitiser NOXA-BH3 (Figure 2E) indicating that endogenous pro-apoptotic proteins released from BCL-2 transfer to MCL-1 and thus render the cells sensitive to the MCL-1 antagonist NOXA. The residual capacity of KG-1a cells triggered with ABT-199 to respond to BAD-BH3 was abrogated when ABT-737 (which targets BCL-X_L_ as well as BCL-2) was used as trigger, and this was associated with over-expression of BCL-X_L_ in these cells (Supplementary Figure 2), likely providing an additional target for BAD-BH3. Strikingly, the TG02/BAD-BH3 and ABT-199/NOXA-BH3 combinations were effective except in the case of OCI-AML3's failure to respond to the ABT-199/NOXA-BH3 combination. As these cells were also insensitive to killing by ABT-199 (Figure 2B), this suggested that only effective drugs would be complemented by the appropriate BH3 peptide and therefore the study was expanded, as described below.

### Effects of TG02 AND ABT-199 in primary samples

**Figure 3:**
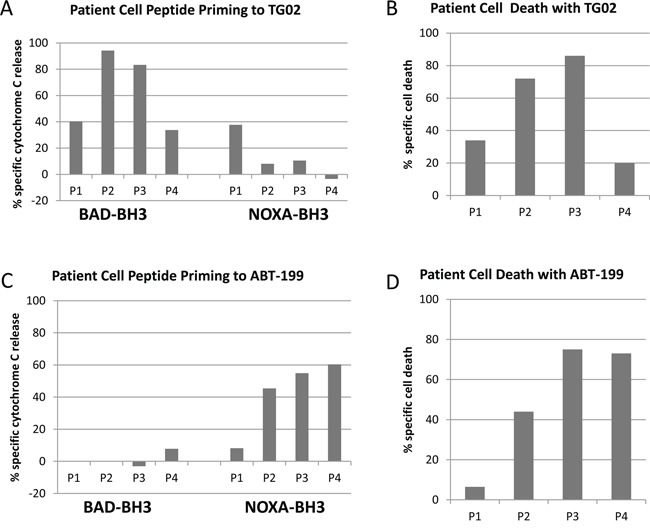
Dynamic BH3 responses in primary samples Four patient samples were treated with 100 nM TG02 **A, B**. or 100 nM ABT-199 **C, D**. After 4 hours, cells were permeabilised and treated for 1 hour with 100 μM NOXA-BH3 or 0.3 μM BAD-BH3, before fixation and labelling with Cytochrome C antibody. A, C, BAD BH3 and NOXA BH3 bars show the percentage of cells which had released cytochrome C. Adjustments for peptide-induced cytochrome C release in untreated cells were made by calculating agent-specific release according to the formula in the Methods section. B, D, Toxicity bars refer to percentage loss of control viable cell number of each agent after 16-20 hours’ culture with drugs at 25 nM, relative to untreated cell number, measured by flow cytometry with 7-AAD and an internal standard for viable cell counting.

To further explore the general applicability of complementary profiling, and whether the apparent correlation between priming and cell killing could be extended to primary cells, we turned our attention to samples from AML patients. Dynamic BH3 profiling using BAD-BH3 and NOXA-BH3 peptides illustrated that the preference for TG02 to sensitise to BAD and of ABT-199 to sensitise to NOXA, shown above in AML cell lines, is also true of patient cells (Figure 3). As with the cell lines, the main implication is that there is residual capacity in these primary samples targeted by one agent to respond to a peptide antagonising a complementary survival pathway. The cytotoxicity of the drugs corresponded closely to the degree of complementary peptide priming. The example of sample P1 in Figure 3 (low cytotoxicity of ABT-199 coupled with low NOXA-BH3 priming, as with the OCI-AML-3 cells) suggests that, at the concentrations used, the peptides are not triggering MOMP in their own right, but prime the cells to release Cytochrome C in the presence of an appropriate trigger of a complementary pathway.

### TG02 and ABT-199 synergise to induce apoptosis

**Figure 4:**
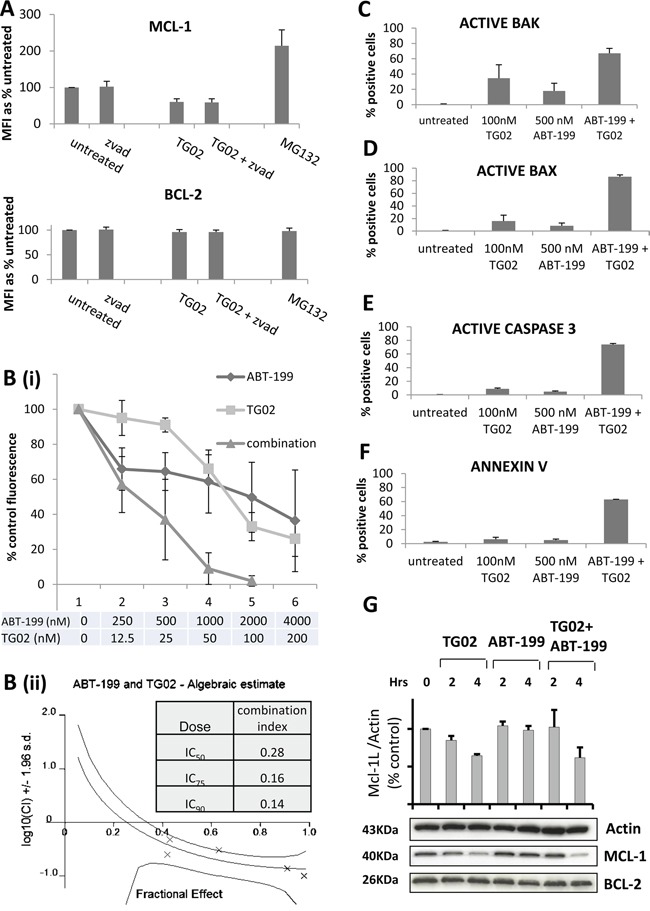
The combination of TG02 and ABT-199 synergises to induce apoptosis in KG-1a cells **A**. KG-1a cells were treated for 45 minutes with 10 μM Z-VAD or 0.5 μM MG132 and for a further 4 hours with 100 nM TG02. Alterations in total MCL-1 and BCL-2 expression were measured by flow cytometry. MFI = mean fluorescence intensity. **B**. KG-1a cells were incubated for 18-22 hours with TG02 and ABT-199 at a fixed dose ratio of 1(TG02):20(ABT-199). Toxicity was measured by alamar blue. **B(i)**. Dose-response curves; **B(ii)**. shows the combination index distributions generated by the Calcusyn software. **C-F**. KG-1a cells were treated for 16-18 hours with 100 nM TG02 and/or 500 nM ABT-199, and then fixed and permeabilised. (C) Percentage of cells with activated BAK; (D) Percentage of cells with activated BAX; (E) Percentage of cells with activated caspase 3; (F) Percentage of Annexin V positive cells. All experimental results (A-F) are mean ± SD of 3-4 independent assays. **G**. KG1a cells were treated with 100 nM TG02 and/or ABT-199 for the indicated times and analysed for MCL-1 and BCL-2 by Western Blotting. The band at 40 kDa represents the long (anti-apoptotic) variant of MCL-1. The barchart shows MCL-1_L_: actin ratios calculated by densitometry (for n=2 independent assays).

The dynamic profiling data demonstrate that, when MCL-1 is targeted, the cells primed with BAD undergo MOMP and when BCL-2 is targeted, the cells primed with NOXA undergo MOMP. This shows that the cells have residual capacity, when targeted by one agent, to respond to a complementary pathway, and further suggests that TG02 might co-operate with ABT-199 in cells that are dependent on both BCL-2 and MCL-1. As preliminary investigation had shown limited efficacy of these compounds individually in KG-1a cells, this cell line was selected for analysis of a combinatorial approach. KG-1a are multidrug resistant, CD34+, CD38 +/- cells which express negative-to low levels of BCL2A1 and BCL2L10 (data not shown); thus MCL-1 is the only one of the three NOXA-targeting labile BCL-2 family pro-survival proteins to be significantly expressed in these cells. After 4 hours of treatment, TG02 down-regulates MCL-1 (by 40%) but not BCL-2 (Figure 4A). The use of the caspase inhibitor Z-VAD indicates that the loss of MCL-1 precedes caspase activation. The use of the proteasome inhibitor MG132 confirms that, in this cell line, MCL-1 protein expression has the expected high dependence on proteasome-mediated degradation [23] (contributing to its short half-life) and we show the contrast with BCL-2, which is not subject to similar proteasomal degradation.

In the KG-1a cells, synergy was documented for the combination of TG02 with ABT-199, (Figure 4B). Briefly, using the median effects method of Chou and Talalay, a combination index (CI) of around 1.0 shows an additive relationship. Antagonism increases with increasing CI above 1.0 and synergy is reflected in a decreasing CI [24]. In the current study strongly synergistic combinations are characterised by low CIs, evident across several concentrations, decreasing with dose and reaching as low as 0.14 at the higher concentrations associated with cytotoxic (rather than/as well as cytostatic) effects. A series of additional assays suggested largely cytostatic effects for the single agents and confirmed that the combination induces apoptotic cell death (Figure 4C-4F). BAK and BAX are important components of the mitochondrial apoptotic pathway, through their ability to create oligomeric pores in the mitochondrial outer membrane [1]. Exposure of activation-sensitive epitopes of BAK and BAX, measured by antibodies specific for activated conformations [25, 26], has previously been described in sensitive cells following exposure to the dual BCL-2/BCL-X_L_ inhibitor ABT-737 [27]. Moreover depletion of BAX or BAK confers resistance to TG02 [28] and ABT-737 [11], although ABT-199 has not been reported in this context. We found stronger induction of BAK activated conformation in cells treated with combinations compared to single agent treatment (Figure 4C), We also found strong induction of BAX activated conformation in cells treated with combinations, contrasted with single digit effects with single agent treatment (Figure 4D). Active caspase 3 and phosphatidylserine exposure were induced strongly by the combination, but weakly by the single agents (Figure 4E and 4F), indicative of largely growth-inhibitory effects of the agents used individually and apoptotic effects for the combination. In a control experiment, the caspase inhibitor Z-VAD did not inhibit BAX or BAK activation, but blocked caspase 3 activation (data not shown). We further showed that the downregulation of MCL-1, previously noted with TG02 alone, was delayed at 2 hours but unimpaired at 4 hours of the drug combination, although ABT-199 alone had no effect on this protein (Figure 4G).

**Figure 5:**
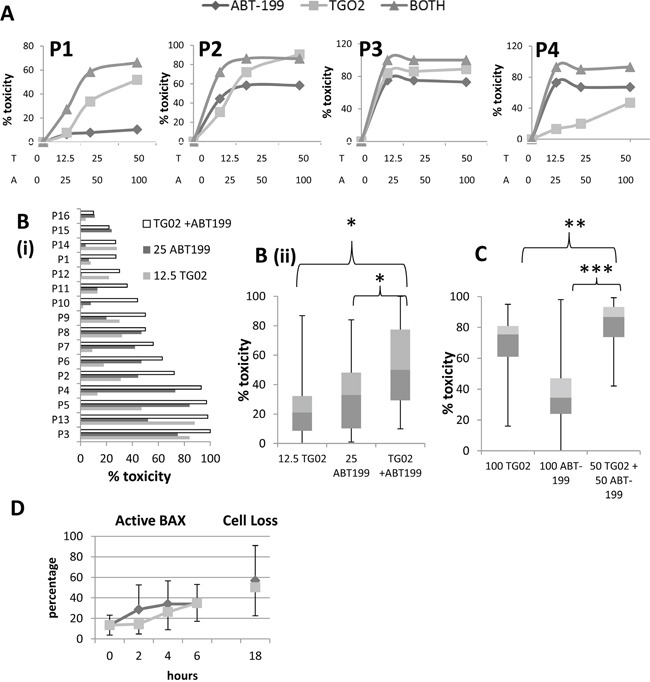
The combination of TG02 and ABT-199 in patient cells **A**. 16-20 hour responses to a range of concentrations of TG02, ABT-199 and the combination were measured by flow cytometric viable cell counting in the same four patient samples as had been used to generate Figure 3. The combination was used in the ratio TG02:ABT-199 1:2. X-axis labels T = nM TG02, A = nM ABT-199. **B**. 16 primary AML samples (including P1 to P4, as in Figures 3 and 5A) were treated for 16-20 hours *in vitro* with 12.5 nM TG02, 25 nM ABT-199 or the combination. Cytotoxicity was determined by flow cytometric viable cell counting. **B(i)**. individual samples. **B(ii)**. summary: median, interquartile range (boxes) and range (error bars) of 18 samples. * denotes P=0.001 in the Wilcoxon signed rank test. **C**. 18 primary AML samples were treated for 16-20 hours *in vitro* with 50 nM TG02 + 50 nM ABT-199 or 100 nM of the agents individually. Toxicity was determined by flow cytometric viable cell counting. The summary graph shows percentage decrease in viable cells. Median, interquartile range (boxes) and range (error bars) of 18 samples. ** denotes P=0.02, *** denotes P<0.001 in the Wilcoxon signed rank test. **D**. Active Bax was measured by flow cytometry at early timepoints after treatment with TG02 (pale grey squares) or ABT-199 (dark grey diamonds) (Mean + standard deviation for 4 samples). Cytotoxicity at 16-20 hours (marked 18 on the X axis) is also shown.

Initial experiments comparing responses to TG02, ABT-199 and the combination in patient samples showed a wide range of responsiveness, as represented in Figure 5A. Some samples responded strongly to TG02, others to ABT-199 and some to both drugs, such that it was not appropriate to standardise a dose ratio and thus formally assess synergy in this cohort. However, we investigated the toxicity of the ABT-199 and TG02 combination in primary samples treated overnight with both low doses and high doses of the agents singly and in combination. With low doses - 12.5 nM TG02, 25 nM ABT-199, or the combination, we found the combination to be significantly more toxic than either agent individually (P=0.001 for both agents, Figure 5B). Some samples were markedly more sensitive to one agent than the other, and other samples were sensitive to both, indicating that in this heterogeneous disease, the combinatorial approach broadens the range of samples that can be effectively killed.

It might be argued that the effects of apoptosis-inducing agents should be studied at high concentrations in order to document their abilities to eradicate the cells, so we also treated 18 primary samples with the higher concentrations of 50 nM TG02 + 50 nM ABT-199 or 100 nM of the 2 agents individually. We showed that using either agent at 100 nM was overall less effective than the combination of 50 nM of each drug (Figure 5C). Another point of note is that whereas the toxicity of ABT-199 reaches a plateau at around 25 nM, the toxicity of TG02 continues to increase, and the majority of cells are eradicated at 100 nM, as previously reported [17].

Active BAX was measured at early timepoints and showed that BAX activation can be seen as early as 2 hours post ABT-199 treatment in sensitive samples and 4 hours with TG02 (Figure 5D).

**Figure 6:**
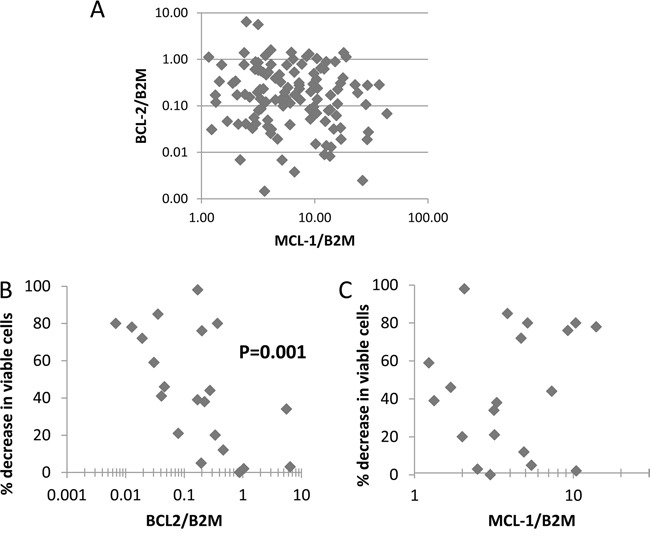
Primary cell survival in TG02-treated cells against BCL-2 and MCL-1 expression **A**. Basal expression of BCL-2 and MCL-1 relative to the housekeeping gene B2M measured by realtime PCR in 117 AML samples. **B**. BCL-2 and **C**., MCL-1 in samples for which the 48 hour *in vitro* response to TG02 had previously been determined [17].

A combinatorial approach might be less therapeutically valuable if AML patient samples could be grouped into discrete groups of BCL-2 over-expressers and MCL-1 over-expressers, thereby stratifying this population into subsets potentially responsive to each drug as a single agent. We measured BCL-2 and MCL-1 using RNA from 117 CD2+cell-depleted primary samples and show here that there is no trend for primary AML samples to group into distinct MCL1-over-expressing and BCL-2-overexpressing samples (Spearman's rho = -0.11, not significant, Figure 6A). A similar lack of association has been reported by others at the protein level [29]. We have previously reported that sensitivity to TG02 varied among primary AML isolates [17]. We have now measured MCL-1 and BCL-2 in cells from this historical cohort and found that BCL-2 was associated with TG02 resistance (Figure 6B). There was no correlation between MCL-1 expression and TG02 sensitivity (Figure 6C).

## DISCUSSION

BH3 sensitiser molecules BAD and NOXA co-operate to induce apoptosis [10], as do NOXA and the BAD mimetic ABT-737 [30]. We determined the applicability of the BAD/NOXA co-operating interaction to two novel chemotherapeutic compounds, TG02 and ABT-199, in Acute Myeloid Leukaemia. We used dynamic BH3 profiling – a methodology for dissecting mechanisms of apoptosis induction at the functional level through monitoring changes in the abilities of specific pro-apoptotic BH3 peptides to induce cytochrome C release following effective drug priming for cell death [19]. Cells treated for as little as 4 hours with TG02 had enhanced sensitivity to BAD-BH3 but not NOXA-BH3 peptides (see Figure 1 for schematic diagram), suggesting that TG02 indeed acts in a pro-apoptotic manner that is complementary to BAD, most probably due to the depletion of MCL-1. We further showed that cells treated with ABT-199 were sensitive to exogenous NOXA. This shows that there is residual capacity in AML cells to allow triggering of the apoptotic response by priming a complementary pathway (as in Figure 1). KG-1a and MV4.11 cells also retained some capacity to respond to BAD-BH3, likely due to residual BCL-X_L_, which is targeted by BAD, but not by ABT-199. Further work is in progress in our laboratory to determine which other AML drugs have a “NOXA-like” profile, enhancing sensitivity to BAD, and which have a “BAD-like” profile, enhancing sensitivity to NOXA.

We have previously shown that inhibition of RPIIS2 by TG02 strongly correlates with BAX activation in patient samples [18], indicating that the ability of TG02 to induce apoptosis is predicated on its ability to inhibit the CDK9-RPIIS2 axis. The downregulation of MCL-1 and other short half-life proteins by RPII inhibition is well documented [31, 32]. The lack of correlation between MCL-1 expression and TG02 sensitivity in patient samples might be explained by these indirect and variable effects. TG02 has a wide range of potential mediators of its anti-tumour activity [16], and we do not assume that MCL-1 is the single mechanistic target for TG02. However, the results of this report, particularly the sensitisation to BAD-BH3, but not to NOXA-BH3, indicate that targeting MCL-1 is highly relevant to its mode of action. In contrast, the BAD mimetic ABT-199, which specifically targets BCL-2, induced the activation of BAX within two hours in sensitive patient cells, consistent with the direct inhibitory mode of action of this drug. As yet ABT-199 is not as thoroughly characterised as its first generation analogue ABT-737, but many of its interactions are likely to be similar. Resistance to ABT-737 in AML cells can be reversed by MCL-1 knockdown [11, 12]. Moreover the ABT-737 sensitivity of AML samples *in vitro* corresponds to their sensitivity to peptides derived from the BAD BH3 domain, which binds BCL-2, but not those derived from the NOXA BH3 domain (which binds MCL-1) [4].

Intriguingly, whereas resistance to TG02 in primary AML samples is associated with high BCL-2 expression (as shown in Figure 6), others have shown that resistance to ABT-199 in primary AML samples is associated with low BCL-2 expression [33]. The rationale behind a requirement for high BCL-2 for BAD mimetic effects has emerged from experiments in which ABT-737 preferentially targets BCL-2 complexed to the activator BH3 protein BIM, releasing BIM from BCL-2 and subsequently activating BAX [30, 34, 35], thus demonstrating that BCL-2 can act as a reservoir for BH3-only apoptosis activators. The ability of BCL-2, and likely also of MCL-1 [36], to act as reservoirs also helps explain why cellular sensitivity and expression levels do not necessarily correlate.

In the KG-1a cell line, we demonstrated synergy for TG02 with ABT-199. This was greatest at higher doses, and the data in Figure 4 show that the combination was able to effect total eradication of the cells, whereas apoptosis was only weakly induced by the single agents. After a partial response to a low dose of ABT-199, the curve for this agent appeared to reach a plateau, also seen in the patient samples in Figure 5A, attesting well to the high potency and specificity of this compound.

AML patient samples are heterogeneous in their responses to both agents, and the complementarity of the combination may be underpinned by synergy in some cases, but also by broadening the proportion of samples that respond. It is of note that we could reduce the concentration of each compound by 50% and still have a combination that was more effective than either agent alone (Figure 5C). The heterogeneity of MCL-1 and BCL-2 expression in primary samples, and likely even within individual patient isolates, suggests that it would be difficult to establish cut-off points that would enable the clinician to select patients for either MCL-1 or for BCL-2 targeting, and therefore the use of complementary agents may offer an ideal solution to this dilemma. ABT-199 and TG02 are both currently in clinical development in multiple hematologic cancers and the current work establishes a strong rationale for the clinical assessment of the two agents in combination. Other agents targeting BCL-2 and MCL-1 have been discussed in recent reviews [15, 36].

We conclude that TG02 and ABT-199 are complementary in AML. We further conclude that Dynamic BH3 Profiling, a term recently coined by Montero, Letai and co-workers [19], is a powerful technique for early prediction of drug sensitivity in primary samples, and may be transferrable to specialist diagnostic laboratories, as evidenced by publications analysing basal BH3 profiles of AML patient cells [29, 37]. We have shown that the technique may also be used to predict complementarity at the level of apoptosis induction. This technique is therefore likely to have further widespread applicability.

## MATERIALS AND METHODS

### Patient cells: ethics statement

The investigation was conducted on samples obtained with informed consent in accordance with the ethical standards and according to the Declaration of Helsinki and according to national and international guidelines and has been approved by the authors' institutional review board.

### Patient cells: preparation

Mononuclear cells were obtained by standard methods from bone marrow or peripheral blood samples of patients with AML. Cells were cryopreserved until use. Only samples with >90% post-thaw viability were assayed.

### Reagents

TG02 was obtained as a citrate salt from Tragara Pharmaceuticals (San Diego, USA).

ABT-199 was from Active BioChem (Hong-Kong). Interleukin-3 (IL-3) was a gift from Novartis (Basel, Switzerland). Interleukin-6 (IL-6) and stem cell factor and Annexin V were from R&D Systems (Abingdon, UK), thrombopoietin and stromal cell-derived factor 1 from Peprotech (London, UK). Granulocyte colony stimulating factor (G-CSF, Neupogen) was a gift from Amgen. Antibodies used for flow cytometry were active BAX (Clone 3), active caspase 3 PE, Cytochrome C Alexa 647 (#558709), CD34-PerCP and CD38-APC or CD38-PE from Becton Dickinson (Cowley, UK); active BAK (clone TC100/ab1, Millipore, UK), BCL-X_L_ (#2767) from Cell Signalling Technologies (UK distributor New England Biolabs, Hitchin, UK); MCL-1 (#31948) was from Abcam (Cambridge, UK); BCL-2-FITC (Dako #F7053) was from UK distributor Alere (Stockport, UK). For Western blotting, rabbit polyclonal MCL-1 (S-19) (sc-819), mouse monoclonal BCL-2 (C-2) (sc-7382), goat anti-rabbit IgG-HRP (sc-2054) and goat anti-mouse IgG-HRP (sc-2055), were all obtained from Santa Cruz UK supplier Insight Biotechnology, Wembley). CD2 Dynabeads were from Invitrogen (Paisley, UK). z-VAD-fmk was from Calbiochem, Nottingham, UK. Additional reagents were obtained from Sigma (Poole, UK) unless otherwise stated.

### Cell lines and culture

The OCI-AML3 myeloid leukemia cell line was obtained from the German Collection of Microorganisms and Cell Cultures (DSMZ, Braunschweig, Germany). The KG-1a cell line was from the European Collection of Animal Cell Cultures (Salisbury, UK). The MV4.11 cell line was from the American Tissue Culture Collection (Manassas, USA). OCI-AML3 and MV4.11 cell lines were maintained in R10 medium and KG-1a cells in R20 medium (i.e. RPMI 1640 medium with 10% (R10) or 20% (R20) foetal calf serum (FCS; First Link, Birmingham, UK), and 2mM L-glutamine. All cultures were kept at 37°C in 5% CO_2_ and all experiments were performed with cell lines in log phase. Continued testing to authenticate these cell lines was performed using multiplex short tandem repeat analysis (Powerplex 16, Promega, Southampton, UK). Mycoplasma testing was carried out routinely using the Mycoalert mycoplasma detection kit (Lonza, Rockland, USA) and following the manufacturer's instructions.

### Cell line toxicity assays

Cells were set up at 5 X 10^5^/ml and cultured in R10 or R20 (see above) for 18-22 hours. Toxicity was measured using Alamar Blue (Roche, Mannheim Germany) according to manufacturers’ instructions.

### Patient cell toxicity assays

Cells were cultured for 18-22 hours in R10 with 20 ng/ml each of IL-3, IL-6, stem cell factor and 25 ng/ml G-CSF, before analysis by flow cytometric viable cell counting using 7-amino actinomicin D (7-AAD) and fixed cells as internal standard for quantitation as previously reported [17].

### Protein measurement

Protein concentrations were measured by flow cytometry and Western blotting.

#### Flow cytometry

Protein expression of MCL-1 (Abcam #31948) and BCL-2 (Ancell #357-040) were measured by flow cytometry as reported [17].

#### Western blotting

Cells were washed in ice cold PBS and resuspended in lysis buffer (50nM Tris{pH 7.4}), 150mM NaCL (Fisher Scientific, Loughborough, UK), 1% NP-40 (BDH Laboratory supplies, Lutterworth, UK), 0.25% Na-deoxycholate, 1mM EDTA, 2μg/ml leupeptin, 5μg/ml aprotinin, 1μg/ml pepstatin, 20mM NaF, 1mM PMSF and 3mM sodium orthovanadate for 30 minutes. Samples were then sonicated before addition of 200mM PMSF and incubation for 30 minutes on ice. The protein content in the lysate was determined using Bio-Rad dye reagent and resolved by SDS-PAGE. After transfer to a nitrocellulose membrane and blocking in 5% non-fat milk, immunoblotting was carried out with the antibodies described above. Proteins were visualized using chemiluminescence (Hyperfilm ECL; Amersham). The intensity of the signal was analyzed by Adobe Photoshop CC 2014 software.

### BH3 profiling

Cytochrome C release was measured by flow cytometry after incubation of digitonin-permeabilised cells with BH3 peptides as described [33]. BH3 peptides were synthesised by GenScript using published sequences [21]. Reactivities were temperature dependent and were carried out at 23-25°C. Results in patient cells were only deemed valid where cell viability as determined by cytochrome C release in the presence of the mutated BH3 peptide Puma2A (100 μM) was less than 10% and release of cytochrome C in the presence of 10 μM BIM-BH3 as positive control was greater than 90%. In patient cell analysis CD45/side-scatter gating was used to exclude lymphocytes. Adjustments for peptide induced cytochrome C release in untreated cells were made in order to establish agent-specific release, using the formula 100*(release with agent – release without agent)/(100 – release without agent).

### Detection of apoptotic pathways

Active BAX was measured using the Transduction labs clone 3 BAX antibody which recognises a conformationally active epitope [9, 26] in cells which had been fixed and permeabilised using the Leucoperm kit from AbD Serotec. Leucoperm was also used to permeabilise cells for active caspase 3 measurement. Active BAK (clone TC100/ab1, [38]) was measured in cells fixed in 0.25% formaldehyde and permeabilised with 0.05% digitonin as previously reported [39].

### Statistics

Statistics were carried out using SPSS version 21 software (Chicago, IL, USA). Univariate analysis of variance was used for analysis of cell lines. Where patient data was seen to be non-parametrically distributed it was analysed accordingly, i.e with Wilcoxon signed rank tests or Mann Whitney analysis. P values <0.05 were considered statistically significant.

### Synergy calculations

Synergy was determined using Calcusyn Software to perform Chou and Talalay analysis [24] for interactions between compounds.

### mRNA measurement

MCL-1, BCL-2 and beta 2 microglobulin (B2M) expression were measured by realtime PCR of RNA prepared from CD2-depleted samples from untreated patients exactly as previously reported [17].

FB participated in the design of the study, edited the manuscript and contributed TG02.

JR participated in the design of the study and edited the manuscript.

CS performed, oversaw and analysed experiments and edited the manuscript.

AAA performed and analysed experiments.

JM participated in the design of the study and edited the manuscript.

AL participated in the design of the study and edited the manuscript.

NR participated in the design and co-ordination of the study and contributed primary AML samples.

All authors read and approved the final manuscript.

## SUPPLEMENTARY MATERIALS FIGURES AND TABLES


