# Supplementary material for: Complementary dynamic BH3 profiles predict co-operativity between the multi-kinase inhibitor TG02 and the BH3 mimetic ABT-199 in acute myeloid leukaemia cells
Source: Oncotarget. 2016 Apr 15;8(10):16220–32. doi: 10.18632/oncotarget.8742 (PMC5369958; doi:10.18632/oncotarget.8742)
Supplement: Supplementary file 1 [file oncotarget-08-16220-s001.pdf]

SUPPLEMENTARY FIGURES

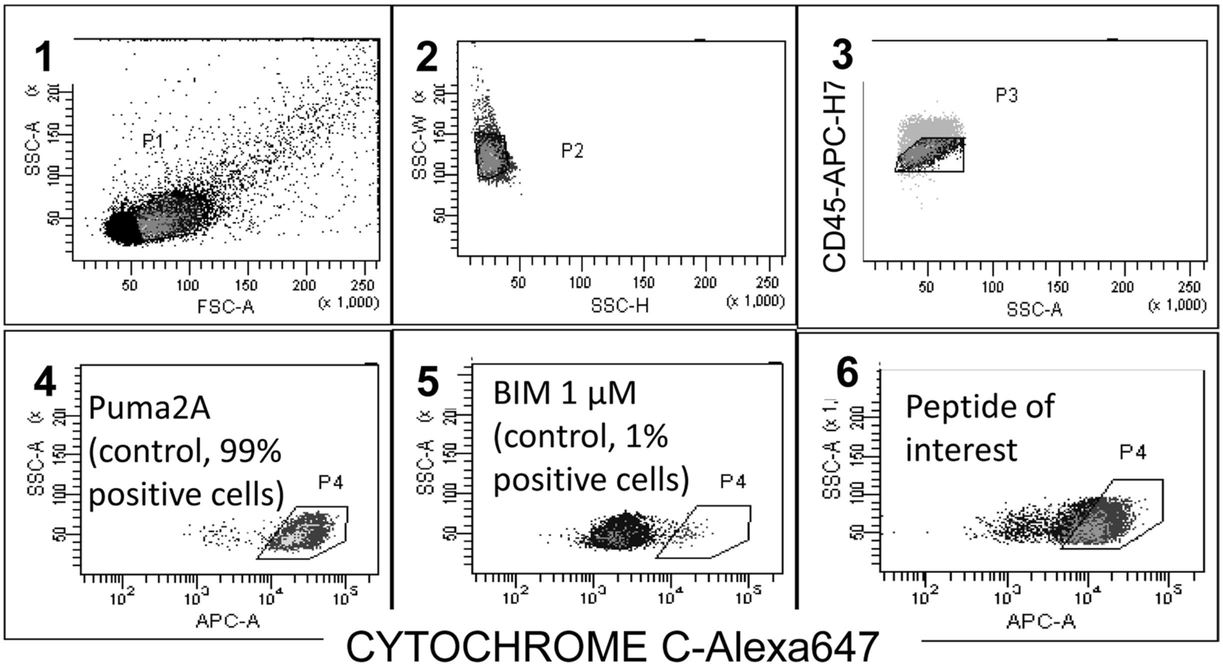

Supplementary Figure S1: Flow cytometry schema for BH3 profiling. Viable cells with effective digitonin permeabilisation are crucial for the BH3 profiling assay. To address this, in addition to conventional FSC/SSC gating (plot 1) and SSC/CD45-gating (plot 3, patient cells only), we gate tightly on SSC height and width to exclude debris (plot 2) and treat digitonin-permeabilised cells with a scrambled BH3 peptide (Puma 2A, plot 4) and with a high BIM-BH3 concentration (plot 5). Viable cells are positive for cytochrome C and hence give a positive signal with Puma 2A. Efficiently permeabilised cells release Cytochrome C on exposure to a high concentration of BIM and this control allows us to confirm that the cells are permeabilised. The proportion of cytochrome C retained by blasts with a given concentration of BH3 peptide is measured as P4 in Plot 6.

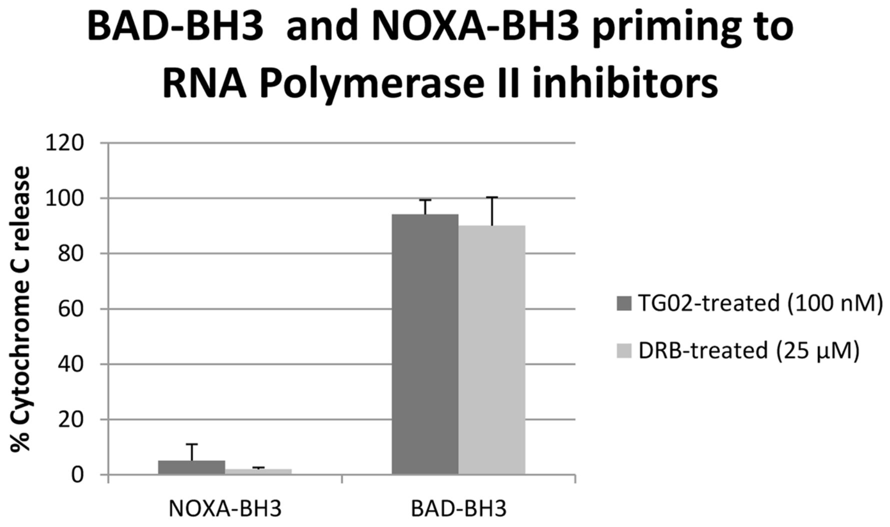

In the BAD-BH3 and NOXA-BH3 assays illustrated in Figure 2D, the specific RNA Polymerase II inhibitor 5,6-dicholoro-1-β-D-ribofuranoslybenzimidazole (DRB) was also investigated and found to have similar effects to TG02.

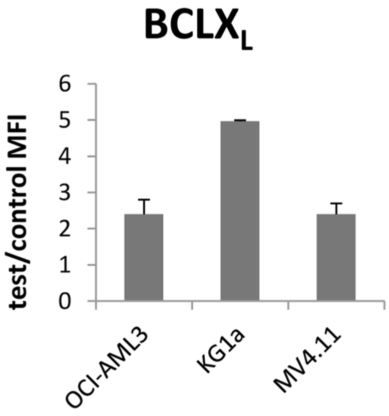

$BCL\text{-}X_L$ was measured by flow cytometry. MFI = mean fluorescence intensity.

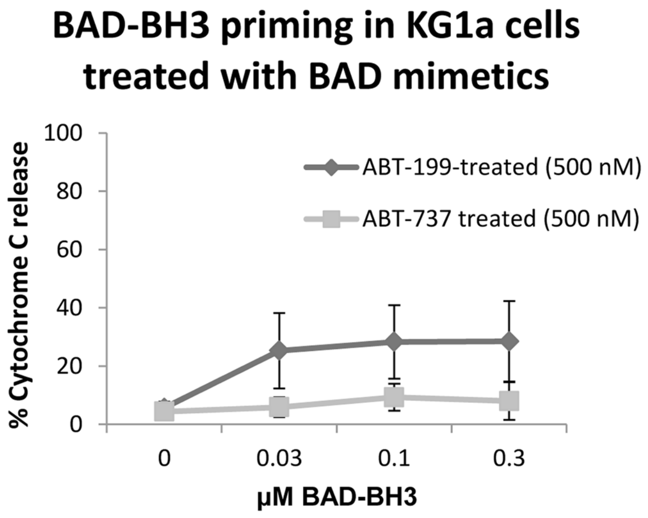

In the BAD-BH3 assay illustrated in Figure 2E, the BAD mimetic ABT-737 which targets $BCL\text{-}X_L$ as well as BCL-2 was used in KG-1a cells and was found not to render the cells sensitive to BAD-BH3 priming.

Supplementary Figure S2: Interactions between BH3 peptides and additional agents.
